# Boosting Natural Killer Cell Therapies in Glioblastoma Multiforme Using Supramolecular Cationic Inhibitors of Heat Shock Protein 90

**DOI:** 10.3389/fmolb.2021.754443

**Published:** 2021-12-01

**Authors:** Tanmoy Saha, Amanda A. van Vliet, Chunxiao Cui, Jorge Jimenez Macias, Arpita Kulkarni, Luu Nhat Pham, Sean Lawler, Jan Spanholtz, Anna-Maria Georgoudaki, Adil Doganay Duru, Aaron Goldman

**Affiliations:** ^1^ Division of Engineering in Medicine, Brigham and Women’s Hospital, Boston, MA, United States; ^2^ Department of Medicine, Harvard Medical School, Boston, MA, United States; ^3^ Glycostem Therapeutics B.V., Oss, Netherlands; ^4^ Xsphera Biosciences Inc., Boston, MA, United States; ^5^ Department of Neurosurgery, Brigham and Women’s Hospital, Boston, MA, United States; ^6^ Department of Neurology, Brigham and Women’s Hospital, Boston, MA, United States; ^7^ Cancer Immunology, Dana Farber/Harvard Cancer Center, Boston, MA, United States

**Keywords:** cell therapy, glioblastoma multiforme, nanotechnology, natural killer cells (NK cells), allogeneic natural killer cells

## Abstract

Allogeneic natural killer (aNK) cell adoptive therapy has the potential to dramatically impact clinical outcomes of glioblastoma multiforme (GBM). However, in order to exert therapeutic activity, NK cells require tumor expression of ligands for activating receptors, such as MHC Class I peptide A/B (MICA/B) and ULBPs. Here, we describe the use of a blood–brain barrier (BBB) permissive supramolecular cationic drug vehicle comprising an inhibitor of the chaperone heat shock protein 90 (Hsp90), which sustains a cytotoxic effect on GBM cells, boosts the expression of MICA/B and ULBPs on the residual population, and augments the activity of clinical-grade aNK cells (GTA002). First, we identify Hsp90 mRNA transcription and gain of function as significantly upregulated in GBM compared to other central nervous system tumors. Through a rational chemical design, we optimize a radicicol supramolecular prodrug containing cationic excipients, SCI-101, which displays >2-fold increase in relative BBB penetration compared to less cationic formulations in organoids, *in vitro*. Using 2D and 3D biological models, we confirm SCI-101 sustains GBM cytotoxicity 72 h after drug removal and induces cell surface MICA/B protein and ULBP mRNA up to 200% in residual tumor cells compared to the naked drug alone without augmenting the shedding of MICA/B, *in vitro*. Finally, we generate and test the sequential administration of SCI-101 with a clinical aNK cell therapy, GTA002, differentiated and expanded from healthy umbilical cord blood CD34^+^ hematopoietic stem cells. Using a longitudinal *in vitro* model, we demonstrate >350% relative cell killing is achieved in SCI-101–treated cell lines compared to vehicle controls. In summary, these data provide a first-of-its-kind BBB-penetrating, long-acting inhibitor of Hsp90 with monotherapy efficacy, which improves response to aNK cells and thus may rapidly alter the treatment paradigm for patients with GBM.

## Introduction

GBM is the most common and aggressive primary malignant tumor of the brain and also one of the deadliest malignancies (Ostrom et al., 2016). Over the past several decades, there has been little progress in therapeutic interventions for GBM, which remain the conventional use of chemotherapy, radiation, and maximum surgical resection (Fernandes et al., 2017). One of the contributions to GBM refractoriness is the relative lack of immunogenicity, defects in T-cell responses, and promotion of an immunosuppressive tumor microenvironment (TME) (Pombo Antunes et al., 2020). In fact, once T-cells penetrate into GBM, they have been observed, *in vivo*, to become dysfunctional via various mechanisms (Woroniecka et al., 2018). For this reason, among others, immune checkpoint blockade and various other immunotherapeutic approaches have proven lackluster in the clinical management of GBM (Weenink et al., 2020).

Molecular chaperones, proteins that play critical roles in proteome homeostasis (Hartl et al., 2011), have been heavily implicated in GBM (Iglesia et al., 2019) and are separately known to play a major role in immune response and immunology (Young et al., 1993; Calderwood et al., 2016). However, the intersection of these two phenomena and tractable therapeutic strategies remains ambiguous and understudied. For example, heat shock protein 90 (Hsp90), an ATP-dependent dimeric molecular chaperone, which forms the core of large complexes with co-chaperones and substrates (Zhao et al., 2005) has been implicated in both pro-inflammatory and anticancer phenotypes as well as functioning as an antagonist of immune-modulating checkpoint inhibitors, which lead to diminished anticancer efficacy (Proia and Kaufmann, 2015). While there are ongoing pre-clinical studies to identify effective Hsp90 inhibitors for GBM (Chen et al., 2020), achieving effective and non-toxic strategies that also augment immunogenicity remains an unaddressed challenge.

Immunotherapy for cancer is composed of a diversity of strategies and modalities including the use of manipulated allogeneic and autologous immune cells. While both T-cell and natural killer (NK) cell therapies have shown promise for hematologic cancers, NK cell therapies are showing increasing promise for solid cancers (Veluchamy et al., 2017) including GBM (Burger et al., 2019) owing to their ability for off-the-shelf allogeneic anti-tumor potential with relatively few side effects after transplantation (Albinger et al., 2021). Retargeting NK cells through the expression of activating receptors including the natural killer cell group 2D (NKG2D) can augment anticancer cytolytic effects in solid cancers (Xiao et al., 2019; Sayitoglu et al., 2020). However, there exist challenges and limitations in NK cell therapy, which can be mainly attributed to limited infiltration of NK cells into solid tumors, downregulation of target antigens on the tumor cells, or suppression by the chemokines and secreted factors present within the TME (Mantesso et al., 2020). The integration of engineering and cancer immunotherapy could address these challenges and spur novel discoveries for treatment (Craig et al., 2020).

We recently reported a novel Hsp90 inhibitor nanoparticle using supramolecular nanochemistry to build conjugated prodrugs, which limit systemic uptake and induce NKG2D ligand expression on solid tumors, *in vivo* (e.g., MICA/B) (Smalley et al., 2020). Here, we describe a novel treatment opportunity for GBM using the synthesized cationic supramolecular inhibitor of Hsp90 (termed “SCI-101”), which is developed for optimal crossing of the BBB in organoid models, monotherapy anticancer efficacy in 2D and 3D GBM models, and sustained expression of NK cell–activating target antigens on tumor cells for more than 72 h following treatment cessation. As a result of this boosted ligand expression, we show that clinically deployed aNK cells, which seek out NKG2D ligands, can practically eliminate drug-resistant GBM cells after exposure to SCI-101. These data warrant further *in vivo* studies to establish a novel combination treatment paradigm that could revolutionize NK cell therapy for patients with GBM.

## Materials and Methods

### Cell Lines, Reagents, Chemicals, and Drugs

U-87, U-251, and LN-229 were obtained from the ATCC and cultured in DMEM + 10% fetal bovine serum (FBS). NK-92 and NK92-MI cells (ATCC) were cultured in alpha-MEM (Gibco, with L-glutamine, without ribonucleosides and deoxyribonucleosides) with 12.5% FBS (Corning, regular) and 0.2 mM myo-inositol (Alfa Aesar). All incubation/culture conditions were 37°C and 5% CO_2_. Radicicol was purchased from Cayman Chemical at a purity >95% and stored in a stock solution of DMSO at −20°C. Primary human astrocytes (Lonza Bioscience) were cultured in Astrocyte Growth Medium (AGM; astrocyte basal medium supplemented with human endothelial growth factor, insulin, ascorbic acid, GA-1000 (gentamicin, amphotericin B), L-glutamine, and 1% fetal bovine serum (FBS); Lonza Bioscience). Human brain microvascular pericytes (HBVP; ScienCell Research Laboratories, Carlsbad, CA) were cultured in Pericyte Medium (ScienCell Research Laboratories) containing 2% FBS, pericyte growth supplement, and penicillin–streptomycin. Human cerebral microvascular endothelial cells (ECs) (hCMEC/D3; Cedarlane, Canada) were maintained in culture using Endothelial Growth Medium (EGM-2; Lonza Bioscience) containing human endothelial growth factor, hydrocortisone, GA-1000, FBS, VEGF, hFGF-B, R^3^-IGF-1, ascorbic acid, and heparin (Lonza Bioscience). The chronic myelogenous leukemia cell line K-562 (ATCC) was cultured in Iscove’s Modified Dulbecco’s Medium (Lonza) + 10% fetal bovine serum (Gibco). For GTA002 experiments, human glioblastoma cell lines U-87 MG (HTB-14, ATCC) and U-251 MG (ECACC) were transduced with IncuCyte NucLight Red (NLR) Lentivirus (Essen Bioscience) to stably express the nucleus-restricted red fluorescent protein mKate2. Transduced cells were banked after selection using 1 μg/ml puromycin (Sigma-Aldrich). For GTA002 experiments, U-87 MG was cultured in Roswell Park Memorial Institute 1640 Medium (Lonza) + 10% fetal bovine serum (Gibco) and U-251 MG was cultured in Dulbecco’s Modified Eagle’s Medium (Lonza) + 10% fetal bovine serum (Gibco). All cell lines were cultured at 37°C and 5% CO_2_.

### Bioinformatics From Clinical Datasets

The cBioPortal for *Cancer* Genomics (http://cbioportal.org) platform was queried for this study. mRNA co-expression was determined and quantified by RNA Seq V2 RSEM [log_2_(value + 1)]. Analyses and graphs were generated using cBioPortal online software, or raw values were exported and plotted with GraphPad Prism software. Statistics were performed as described in figure legends.

### RNA Extraction and Quantitative PCR (qPCR)

Following treatment, as described in figure legends, the cells were transferred into 1.5 ml Eppendorf tubes, and the resulting cell pellets were frozen in 1 ml of Trizol (Invitrogen, Cat. No. 15596026) at −80°C for RNA extraction and cDNA synthesis (Invitrogen’s SuperScript VILO cDNA Synthesis Kit, Cat. No. 11754050). cDNA was diluted 1:5 and used for quantitative PCR following manufacturers’ protocol (Applied Biosystems’ PowerUp^TM^ SYBR^TM^ Green Master Mix kit, Cat. No. A25742). Integrated DNA Technologies (IDT) PrimeTime^TM^ predesigned qPCR primers with intercalated dyes were used for all genes analyzed (ULBP1–4, MICA, MICB, HLA-C). The resulting gene expression data were plotted according to the delta–delta CT (ΔΔCT) method, and statistical significances in gene expression were calculated using the two-way ANOVA (Sidak’s multiple-comparison test).

### Microfluidic 3D GBM Spheroid Preparation and NK92-MI Cell Co-Culture Experiments

U-251 and U-87 single cells were seeded in a six-well ultra-low attachment plate (Corning) and cultured for 24 h to generate spheroids. The seeding density for both cell lines was 125k cells/mL, and 5 ml media were added into each well. Spheroids were then filtered through a 100 µm strainer followed by a 40 µm strainer (Falcon) to collect tumor spheroids with sizes between 40 and 100 µm. Tumor spheroid suspensions were pelleted via centrifugation (300 x g) and resuspended in type I rat tail collagen (Corning) at a concentration of 2.8 mg/ml (adjusted by adding 10X PBS, 0.5N NaOH, and H_2_O to reach a pH value between 7.0 and 7.5). The spheroid/collagen mixture was then injected into the center gel channel of an AIM 3D microfluidic cell culture chip (AIM Biotech) followed by 35 min incubation allowing collagen to polymerize. Fresh media were added into the two inner media channels on the AIM chip for hydration and 75 µL media ± SCI-101 at the indicated concentrations. After 24 h, the media were removed and replaced by fresh media for further culturing for an additional 24 h. Where indicated, NK92-MI cells were pre-incubated with the cell permeable fluorescent dye carboxyfluorescein succinimidyl ester (CFSE) at 1 μM and added to the microfluidic port of the AIM chip to achieve an effector:tumor (E:T) ratio of 5:1. The samples were subsequently cultured for three additional days in the presence of NK cells or untreated. At the termination of the experiment, the cytotoxicity of GBM spheroids was analyzed by Hoechst 33342 and propidium iodide staining in media (Thermo Fisher, diluted at 1:800), which was added into the media port and incubated for 45 min before imaging following the previously described protocol (Jenkins et al., 2018). Images were taken using a Nikon Eclipse fluorescence microscope (Nikon), and live/dead staining was quantified by measuring the total cell area of each dye following our previous methodology (Jenkins et al., 2018). Infiltration of NK92-MI cells into the tumor spheroids was determined using CS5 software (Adobe) measuring fluorescent intensity in uniform, defined tumor spheroid regions of interest (ROIs). Relative fluorescence units were determined and plotted for each individual ROI.

### Hematopoietic Stem Cell Isolation and NK Cell Culture

Hematopoietic stem cells, isolated from fresh umbilical cord blood (Anthony Nolan, United Kingdom), were expanded and differentiated into GTA002 NK cells as previously described (Spanholtz et al., 2010). In short, CD34^+^ cells were seeded at a concentration of 10,000 cells/ml in a six-well tissue culture–treated plate (Corning Incorporated) in Glycostem Basal Growth Medium (GBGM^®^, FertiPro) supplemented with 10% human serum (Sanquin, Netherlands), 25 ng/ml of TPO, IL-7, Flt-3L, and SCF (Cellgenix), 1 ng/ml GM-CSF, 0.05 ng/ml Il-6 (Cellgenix), and 0.25 ng/ml Neupogen (G-CSF; Amgen BV). After 9 days, TPO was replaced by 20 ng/ml IL-15 (Cellgenix). After 14 days of expansion, the differentiation phase was initiated by adding differentiation medium consisting of GBGM supplemented with 2% human serum, 20 ng/ml of IL-7, IL-15, and SCF, 1 ng/ml GM-CSF, 0.05 ng/ml Il-6, 0.25 ng/ml Neupogen, and 1000 U/ml Proleukin (IL-2; Novartis). The cells were cultured until day 28 and cryopreserved until further use. For usage in cytotoxicity assays, day 28 GTA002 cells were thawed and cultured for 1 week in differentiation medium.

### Flow Cytometry–Based Cytotoxicity/Degranulation Assay

K-562 cells were washed with PBS and labeled with 5 μM pacific blue succinimidyl ester (PBSE; Thermo Fisher). Per well, 1 × 10^5^ K-562 cells were co-cultured with GTA002 cells at E:T 1:1 in a total volume of 200 µL in a 96-well V-bottom plate. At the start of co-incubation, anti-CD107a PE (H4A3, BioLegend) was added at 1 μg/ml. After 5 h of incubation at 37°C, the cells were transferred to a V-bottom plate and stained with 4 μg/ml 7-AAD (Sigma) and 1 μg/ml anti-CD56 APC-A750 (N901, Beckman Coulter) in 25 μL. Read-out of the cytotoxicity assay was done using Cytoflex LX (Beckman Coulter), and data analysis was done using Kaluza Analysis 2.1 (Beckman Coulter). Killing was determined by the following formula: %cytolysis = 100-[(number of (7-AAD^−^/PBSE^+^) K-562 cells in co-culture/number of (7-AAD^−^/PBSE^+^) K-562 cells alone)*100]. Degranulation was calculated as % Δ degranulation = (% CD107a^+^/CD56^+^/7-AAD^−^ cells in co-culture) – (% CD107a^+^/CD56^+^/7-AAD^−^ cells in NK cells alone).

### Incucyte Cytotoxicity Assay

NLR U-87 and NLR U-251 were pre-incubated with 5 μM SCI-101 for 72 h. After washing, the glioblastoma cell lines were seeded at 5,000 cells/well in a 96-well plate (Corning). After 5 h of adherence, GTA002 day 35 cells were added to the wells in an E:T ratio of 1:1. The red fluorescent signal was imaged every 2 h for 60 h at ×20 magnification by the IncuCyte S3 live-cell analysis system (Essen Bioscience). The results are shown as cell proliferation which displays the red integrated intensity normalized to time point 0 h and relative viability which is calculated as a ratio between the normalized cell proliferation of NK cell therapy–treated targets ± SCI-101 and the normalized proliferation of the corresponding target only control ± SCI-101

### Soluble MHC Class I Peptide A (MICA) ELISA

Human soluble MICA was evaluated using the sandwich-based colorimetric assay kit (catalog: ELH-MICA-2) following the manufacturer’s protocol (RayBiotech). Briefly, U-87 cells were pre-incubated with SCI-101 as described in the figure schematic following a drug washout (24 h exposure). The cell culture supernatant was then collected after 24 or 72 h and analyzed for soluble MICA expression. The standard curve was determined for each experiment, and experimental results were determined based on these quantitative results.

### Flow Cytometry

The cells were cultured as indicated, collected under Accutase dissociation from culture (Semcell.com), washed twice with PBS, and fluorescently labeled with antibodies against MICA/B (6D4, BioLegend) for 30 min at room temperature. The cells were then analyzed by flow cytometry (C6 Accuri Cytometers, Inc., Ann Arbor, MI). Data analysis was performed using FlowJo software (Tree Star, Inc., Ashland, OR) and Accuri cFlow plus software to obtain and confirm the mean fluorescent intensity. Isotype IgG control was used to subtract background noise. To measure receptor expression on GTA002 NK cells, the following antibodies were used: anti-CD45 KO (J33), anti-CD56 APC-A750 (N901), anti-NKG2D PE (ON72), anti-NKp44 (Z231), anti-NKG2A PE (Z199), anti-KIR2DL1 and -KIR2DS1 PE (EB6B) (all from Beckman Coulter), anti-CD3 (VioBlue; BW263/56), anti-DNAM-1 (REA1040) (both from Miltenyi Biotec B.V.), and 7-AAD (Sigma). All antibodies were used at recommended concentrations and 100,000 cells were stained for 15 minutes at room temperature. After washing, data were acquired with Cytoflex LX (Beckman Coulter) and analyzed with Kaluza Analysis 2.1 (Beckman Coulter).

### Generation of Cationic Nanoparticles

AN-01 preparation: For supramolecule synthesis, L-α-phosphatidylcholine (65 mol%), 5 mol% of NBD-PC, and 30 mol% of 1,2-distearoyl-sn-glycero-3-phosphoethanolamine-N-(amino-[polythylene glycol]2000) (DSPE-PEG) were dissolved in 1.0 ml DCM. The solvent was evaporated into a thin and uniform lipid–drug film using a rotary evaporator. The lipid–drug film was then hydrated with 1.0 ml H_2_O for 2 h at 70°C. It was extruded at 70°C using 400 nm and then 200 nm membranes with 100 μL sample volume to obtain ∼230 nm particles. The nanoscale supramolecular therapeutics were further passed to the Sephadex G-50 column to remove free molecular subunits (free AN-01).

AN-02 preparation: For supramolecule synthesis, L-α-phosphatidylcholine (65 mol%), 5 mol% of NBD-PC, and 30 mol% of 1,2-distearoyl-sn-glycero-3-phosphoethanolamine-N-(carboxy-[polythylene glycol]2000) (DSPE-PEG) were dissolved in 1.0 ml DCM. The solvent was evaporated into a thin and uniform lipid–drug film using a rotary evaporator. The lipid–drug film was then hydrated with 1.0 ml H_2_O for 2 h at 70°C. It was extruded at 70°C using 400 nm and then 200 nm membranes with 100 μL sample volume to obtain ∼230 nm particles. The nanoscale supramolecular therapeutics were further passed to the Sephadex G-50 column to remove free molecular subunits (free AN-02).

### SCI-101 Synthesis

Radicicol-cholesterol (Rad-Ch) synthesis: To a clean and dry flask were added radicicol (0.053 mmol), DIPEA (0.212 mmol), EDC·HCl (0.1 mmol), and 5 ml DCM. The reaction mix was stirred for 10 min at room temperature under inert atmosphere. Cholesteryl hemisuccinate (0.08 mmol) was added and the mixture were stirred for 24 h. The reaction was monitored by TLC, and the expected compound was purified by silica gel chromatography, using a gradient of DCM:MeOH (100:0 to 90:10). The final product Rad-Ch was obtained in 65% yield. For supramolecule synthesis, L-α-phosphatidylcholine (60 mol%), 10 mol% of Rad-Ch, and 30 mol% of 1,2-distearoyl-sn-glycero-3-phosphoethanolamine-N-(amino-[polythylene glycol]2000) (DSPE-PEG) were dissolved in 1.0 ml DCM. The solvent was evaporated into a thin and uniform lipid–drug film using a rotary evaporator. The lipid–drug film was then hydrated with 1.0 ml phosphate buffered saline (PBS) for 2 h at 70°C. It was extruded at 70°C using a 400 nm membrane with 100 μL sample volume to obtain ∼230 nm particles. The nanoscale supramolecular therapeutics were further passed to the Sephadex G-50 column to remove free molecular subunits (free Rad-Ch). Drugs incorporated in Rad-Nano were determined using UV-Vis spectroscopy. The incorporation efficiency was determined as the percentage of drug recovered from the nanoscale supramolecular therapeutic fractions compared to the initial loading amount (data from UV-Vis spectroscopy). The mean particle size of the nanoscale supramolecular therapeutics was measured by a dynamic light scattering (DLS) method using Zetasizer Nano ZS90 (Malvern, United Kingdom). The ζ potential was measured using Zetasizer ZS90 with the nanoscale supramolecular therapeutics diluted in water for measurement according to the manufacturer’s manual. The physical stability of nanoscale supramolecular therapeutics was evaluated by measuring changes in the mean particle size and ζ potential during storage condition at 4°C. The particle size and ζ potential were measured as a function of time using Malvern Zetasizer.

### BBB Organoid Model and Permeability Experiments

Blood–brain barrier organoid models were prepared as described previously (Cho et al., 2017). Briefly, sterile 1% agarose (w/v) was prepared by adding 0.5 mg of molecular biology–grade agarose (Bio-Rad) into 50 ml of PBS in a conical flask and boiled in a microwave until completely dissolved. The melted agarose solution was transferred into a sterile tissue culture hood, and 50 μL of the solution was dispensed into each well of a 96-well plate while it was still hot using a multi-channel pipette and allowed to cool/solidify (∼15 min). Primary human astrocytes were resuspended in HBMEC working medium. Per well, 1 × 10^3^ cells of each cell type were seeded onto the agarose gel in a 96-well plate in a 1:1:1 ratio (final volume = 100 μL). The cells were placed in a humidified incubator at 37°C with 5% CO_2_ and 95% natural air for 48–72 h to allow for the assembly of multicellular BBB spheroids. For spheroid formation using the immortalized hCMEC/D3 EC line: hCMEC/D3, astrocytes and HBVP were released by trypsin/EDTA and resuspended in hCMEC/D3 working medium. Per well, 1.5 × 10^3^ cells of each cell type were seeded onto the agarose gel in a 96-well plate in a 1:1:1 ratio, and spheroids were allowed to form as described above. Spheroids were fixed in 3.7% formaldehyde for 30  min, washed three times with PBS, transferred into a Nunc Lab-Tek II thin-glass eight-well chambered cover glass (Thermo Scientific), and imaged under a Zeiss LSM710 confocal microscope. Quantification of spheroid permeability to fluorescent cationic nanoparticles (AN01 and AN02) was performed using ImageJ software (http://imagej.net/Fiji). The mean fluorescence intensity of the core of each spheroid at 80 μm depth was quantified and plotted using GraphPad Prism software (version 7.0).

### LC/MS Experiments

Radicicol and SCI-101–treated BBB organoids (described above) were dissolved in 100% ethanol and sonicated for 5 min, and pellets were removed before submitting for LC/MS analysis. Liquid chromatography mass spectroscopy (LC-MS/MS) samples were analyzed on an Agilent 1260 Infinity LC system interfaced with an Agilent 6120 single quadrupole MS system (Agilent Technologies). Samples were loaded with the volume of 30 L on a Poroshell 120 EC-C18 column (2.1 × 50 mm, particle size of 2.7 m, Agilent Technologies). Samples were separated with a linear gradient mobile phase from 5 to 95% B over 4 min (5–100% B for 1.75 min, 100% B for 1.25 min, 100–5% B for 0.1 min, and 5% B for 0.9 min) at a flow rate of 1000 L/min. Solvent A was 0.1% formic acid in HPLC-graded water. Solvent B was 0.1% formic acid in HPLC-graded acetonitrile. Mass spectra were acquired by an Agilent 6120 single quadrupole MS system with an ion source of atmospheric pressure and electrospray ionization (API-ES) operated in both positive and negative ion modes. The parameter settings were as follows: resolution of 120,000 resolution, scan range of 100–1,000 m/z, fragmentor of 70 m/z, gain of 1, threshold of 150, step size of 0.10, peak-width of 0.100 min, speed of 1,300 u/sec for positive ionization (MSD1), speed of 2,600 u/second for negative ionization (MSD2), and cycle time of 1.68 s with 60% of cycle time for MSD1 and 40% of cycle time for MSD2. Raw data were analyzed by the Agilent OpenLAB CDS ChemStation Edition (rev. C.01.07) software for peak integration, peak alignment, and mass analysis. The calibration curve of radicicol was determined across a series of concentrations.

### Cell Viability and Cytotoxicity Assays

Cell counts were determined using a hemocytometer or cell viability was measured using Cell Counting Kit-8 (CCK-8, Dojindo) where indicated following the manufacturer’s protocol, and absorbance was read at the recommended UV wavelength (450 nm) using a BioTek microplate reader (BioTek Instruments, Inc.).

### Statistics

Statistical analysis was performed using Prism software (GraphPad) by ANOVA followed by Tukey’s post hoc test when values were represented between multiple groups, and unless otherwise noted, two-tailed Student’s t-test was used to identify statistical significance between individual groups.

## Results

### Hsp90 Is a Clinical Biomarker in Glioblastoma Multiforme (GBM), Sensitive to Pharmacologic Inhibition With Radicicol

**FIGURE 1:**
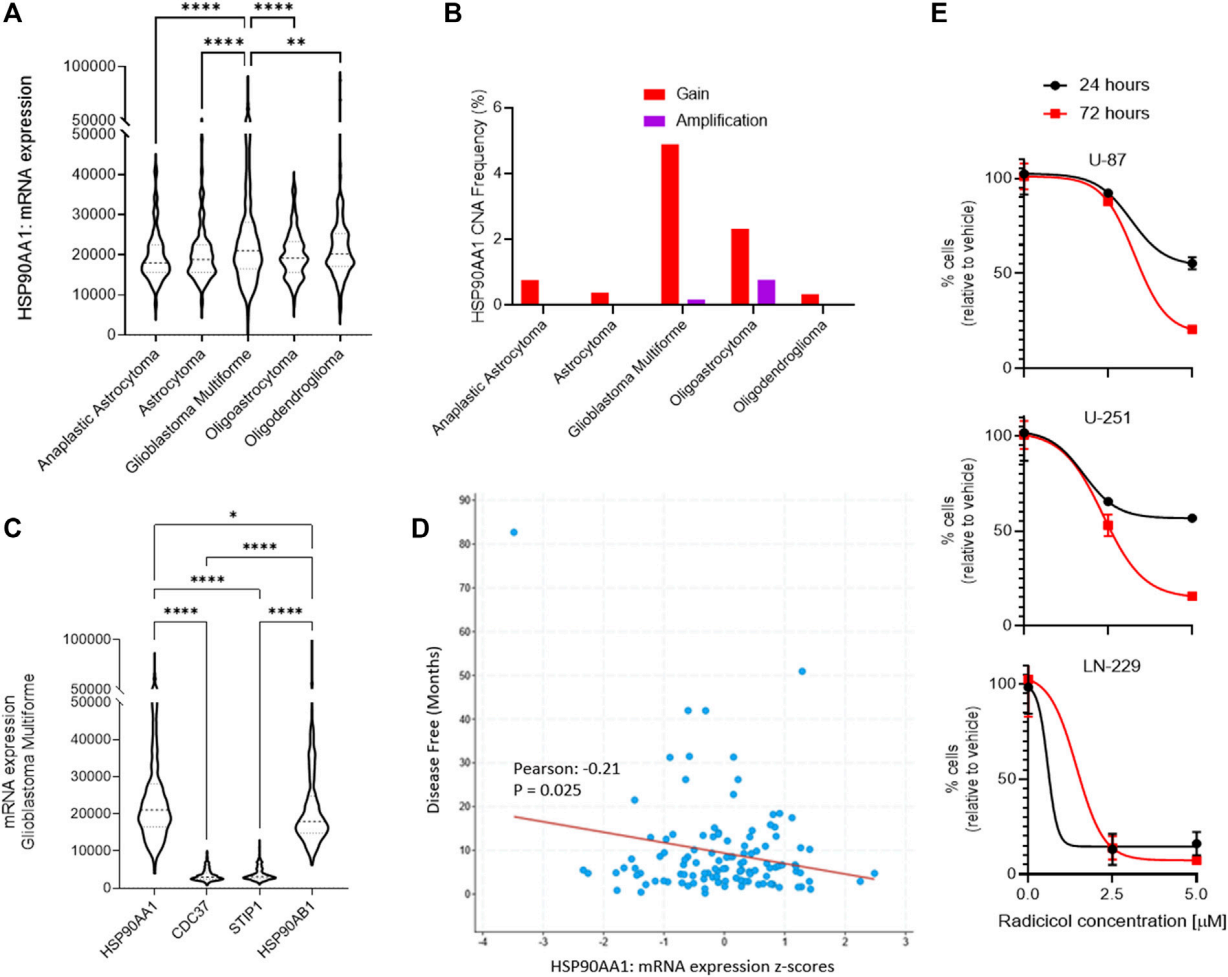
Role of Hsp90 in glioblastoma multiforme (GBM) across CNS tumors. **(A)** HSP90AA1 mRNA expression (RNA Seq V2 RSEM) [log_2_(value + 1)] compared across five clinical studies (cBioPortal). **p<0.01, ****p<0.0001 analyzed by ordinary one-way ANOVA with Tukey’s multiple-comparison test. **(B)** Copy number alteration (CNA) evaluated across seven clinical studies (cBioPortal). **(C)** GBM clinical datasets and TCGA data were queried to compare multiple Hsp90-related chaperone and co-chaperone gene expressions by mRNA. *p<0.05, ****p<0.0001 analyzed by ordinary one-way ANOVA with Tukey’s multiple-comparison test. **(D)** Dot plot comparing disease-free survival with Hsp90 mRNA gene expression from two GBM clinical datasets (cBioPortal). The line indicates simple linear regression. Pearson’s coefficient is statistically significant. **(E)** Cell kill curves of three high-grade glioma/GBM cell lines following treatment with the Hsp90 inhibitor, radicicol, for the indicated amount of time and concentration. N=3 in biological replicate.

Using a bioinformatics approach, we interrogated patterns of Hsp90 gene expression among different central nervous system (CNS) tumors to identify a link to GBM. We queried the open repository of The Cancer Genome Atlas (TCGA) and molecular gene expression data in cBioPortal (CBIOportal.org) and compared CNS tumor subtypes including astrocytoma, GBM, and oligodendrocyte malignancies for the expression of Hsp90 mRNA (HSP90AA1). Among five independent clinical studies comparing >1,500 samples, we determined there is a statistically significant relationship among GBM tumors, compared to all other subtypes, which demonstrate greater overall expression (Figure 1A). Indeed, the combined frequency for Hsp90 gain of expression and gene amplification due to copy number alterations (CNAs) was greatest in the GBM cohorts, which were determined from seven different clinical studies and >3,500 samples (Figure 1B). We then interrogated the mRNA expression of Hsp90 co-chaperones from two GBM clinical datasets, including cell division cycle 37 (CDC37), stress-induced phosphoprotein 1 (STIP1), and Hsp90 class B, demonstrating a clear upregulation of HSP90AA1 in each (Figure 1C). Indeed, disease-free survival and Hsp90 gene expression were inversely correlated in a statistically significant fashion based on Pearson’s correlation coefficient (Figure 1D). Finally, to evaluate therapeutic tractability in GBM models, we applied a small molecule inhibitor of Hsp90, radicicol (Sharma et al., 1998), to multiple GBM-derived human cell cultures with differing published expression levels of Hsp90 from highest to lowest relative to β-actin: LN-229 > U-251 > U-87 (Belkacemi and Hebb, 2014). Each cell line showed time- and dose-dependent sensitivity, which was consistent with the relative Hsp90 expression levels (Figure 1E).

### Synthesis of SCI-101, a BBB Organoid–Penetrating Supramolecular Cationic Inhibitor of Hsp90

**FIGURE 2:**
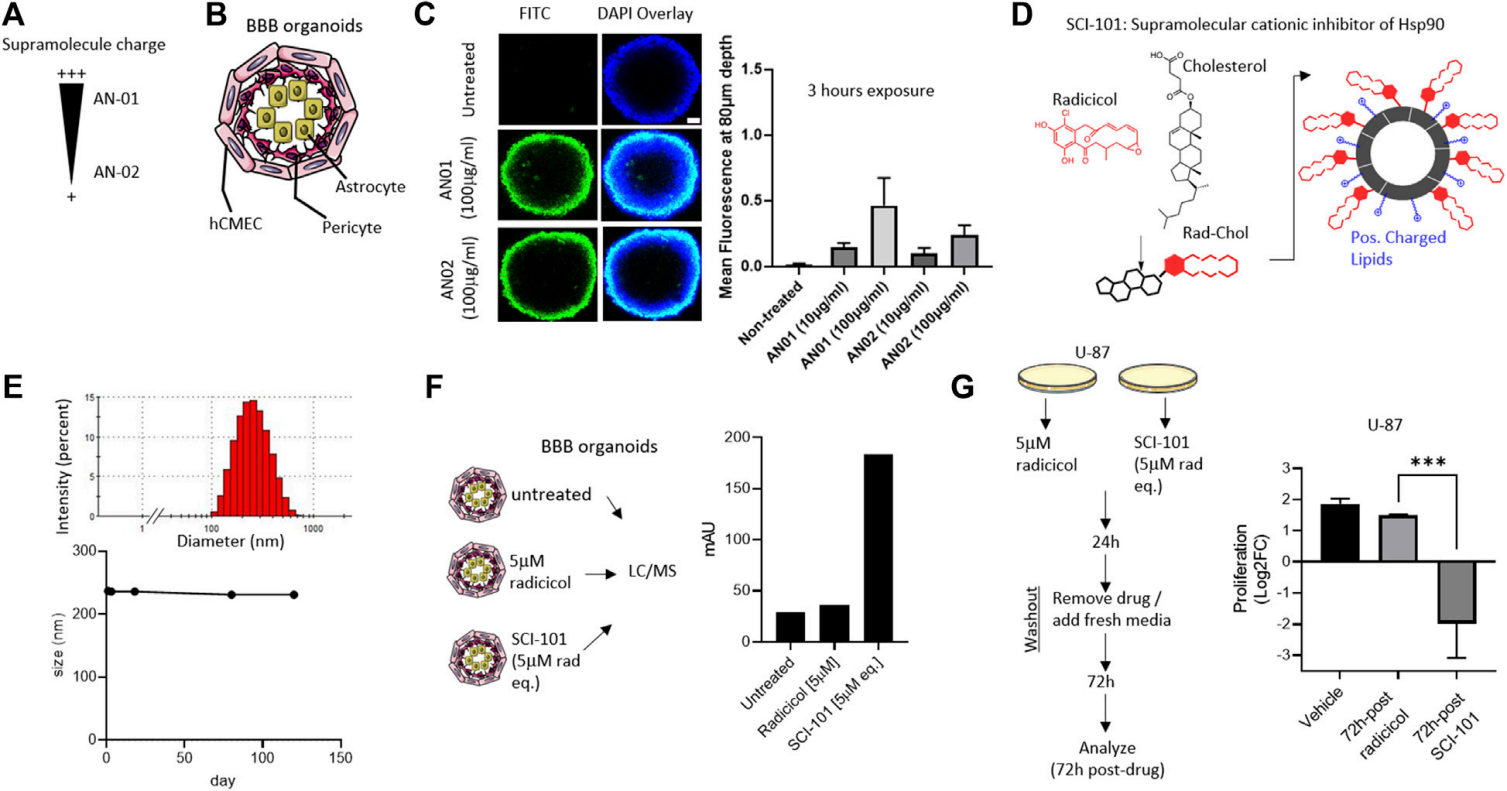
Design, synthesis, and characterization of a BBB organoid–penetrating supramolecular cationic inhibitor of Hsp90. **(A)** The schematic shows the design of supramolecular drug vehicles using lipids with varying headgroup charge: AN01 and AN02. **(B)** The schematic depicts the BBB organoid model used for in vitro experiments. The endothelial cell layer (hCMEC) surrounds the pericyte and astrocyte layers of cells. **(C)** The immunofluorescent image shows infiltration of AN01 and AN02 into the BBB organoid. The green signal indicates the amount of lipid penetrating the endothelial barrier deep enough to account for passage into the BBB organoid (80 μm). Scale bar = 50 μm. The right panel quantifies the fluorescent intensity at a depth of 80 μm. **(D)** The schematic shows the design of SCI-101, comprising cholesterol-radicicol conjugates assembled together with positively charged lipids into a supramolecular structure. **(E)** The representative histogram shows the size distribution of SCI-101 (upper panel) and longitudinal trace of average size over 120 days to demonstrate stability of the drug vehicles (lower panel). **(F)** The schematic describes the experimental design for qualification of radicicol in the BBB organoid. The right panel bar graph quantifies the penetration of radicicol free drug or SCI-101 infiltrated into the BBB organoid as measured by the area under the curve. **(G)** Experimental design schematic for drug washout studies. Following 24 h exposure to either free drug radicicol or SCI-101 at equivalent concentration (5 μm), the cells were washed and recovered with fresh media for 72 h. The right panel histogram quantifies the proliferation of U-87 cells 72 h after drug washout. The values shown are relative to the number of cells prior to drug washout in the vehicle control group. ***p<0.001 by ordinary one-way ANOVA with Tukey’s multiple-comparison test.

Despite promising pre-clinical data, the clinical development of Hsp90 inhibitors capable of avoiding systemic toxicities and penetrating the BBB remains a challenge (Lomeli and Bota, 2018; Putcha et al., 2010). Novel drug carriers can be harnessed to offset toxicity and improve BBB penetration by modulating size, shape, rigidity (Brown et al., 2020), and even surface charge (Lockman et al., 2004). However, no studies have applied these principles to the development of Hsp90 inhibitors and particularly radicicol, which shows excellent pharmacologic properties but was limited by bioavailability, toxicity, and BBB penetrability (Putcha et al., 2010). First, we engineered two supramolecule structures based on variable cationic charges termed “AN01” (more cationic) or “AN02” (less cationic), comprising biocompatible excipients including cholesterol, phosphatidylcholine, and polyethylene glycol (PEG) (Figure 2A). Using a BBB organoid model composed of endothelial, pericyte, and astrocyte layers (Cho et al., 2017), we interrogated the permissiveness of the cationic supramolecules (Figure 2B). Over the course of 3 h, we identified greater penetration into the BBB organoid for AN01, which was determined at a depth into the organoid of 80 microns and sufficiently through the endothelial and pericyte layers quantified by confocal fluorescent intensity in Z-stack (Cho et al., 2017) (Figure 2C). These data are consistent with other reports that interconnect adsorptive-mediated transcytosis or endocytosis with cationic particles (Joshi et al., 2014; Vieira and Gamarra, 2016). Next, we used the optimal cationic properties from AN01 to synthesize a supramolecular cationic inhibitor of Hsp90 comprising radicicol tethered to cholesterol within a positively charged lipid bi-layer (Smalley et al., 2020) and termed this new active pharmaceutical ingredient (API) “SCI-101” (Figure 2D). We confirmed SCI-101 maintains a uniform shape beyond 120 days, as determined by dynamic light scattering (DLS) (Figure 2E).

### Functional Characterization, BBB Organoid Penetration, and Anticancer Effects of SCI-101

Next, we sought to characterize the functional activity of SCI-101 including BBB organoid penetration and anticancer effects vs. the free form drug radicicol. First, we exposed the BBB organoids to either radicicol or the concentration equivalent of SCI-101 for 30 min to 4 h. To quantify BBB organoid permissiveness, we used liquid chromatography in tandem with mass spectrometry (LC/MS-MS) and developed a calibration curve with radicicol (Supplementary Figure 1). In contrast to free-form radicicol, which was undetectable in BBB organoids by LC/MS-MS, SCI-101 incorporated rapidly and sustained over the course of 4 hours to a measurable level as confirmed by mass spectrometry analysis identical to the radicicol reference standard (Figure 2F and Supplementary Figure 2). To test the anticancer efficacy of SCI-101, we developed a translational *in vitro* model to mimic the pharmacokinetics of radicicol or SCI-101. Following the schematic in Figure 2G, we treated U-87 GBM cells with either radicicol or the concentration equivalent of SCI-101 for 24 h followed by drug washout and recovery in fresh media and evaluated endpoints after 72 h of additional culture (total 96 h experiment). We identified that SCI-101 significantly improved longitudinal anticancer activity compared to the free drug, as determined by the change in proliferation of U-87 72 h after drug withdrawal (Figure 2G). In contrast, cells treated with radicicol appeared to recover their proliferative status. These findings are consistent with our previous evidence for rapid and sustained accumulation of supramolecular drug vehicles into cells through active and passive transport mechanisms (Smalley et al., 2020).

### SCI-101 Sustains Induction of Immunogenic Properties on GBM Cells via Upregulation of NKG2D Ligands

**FIGURE 3:**
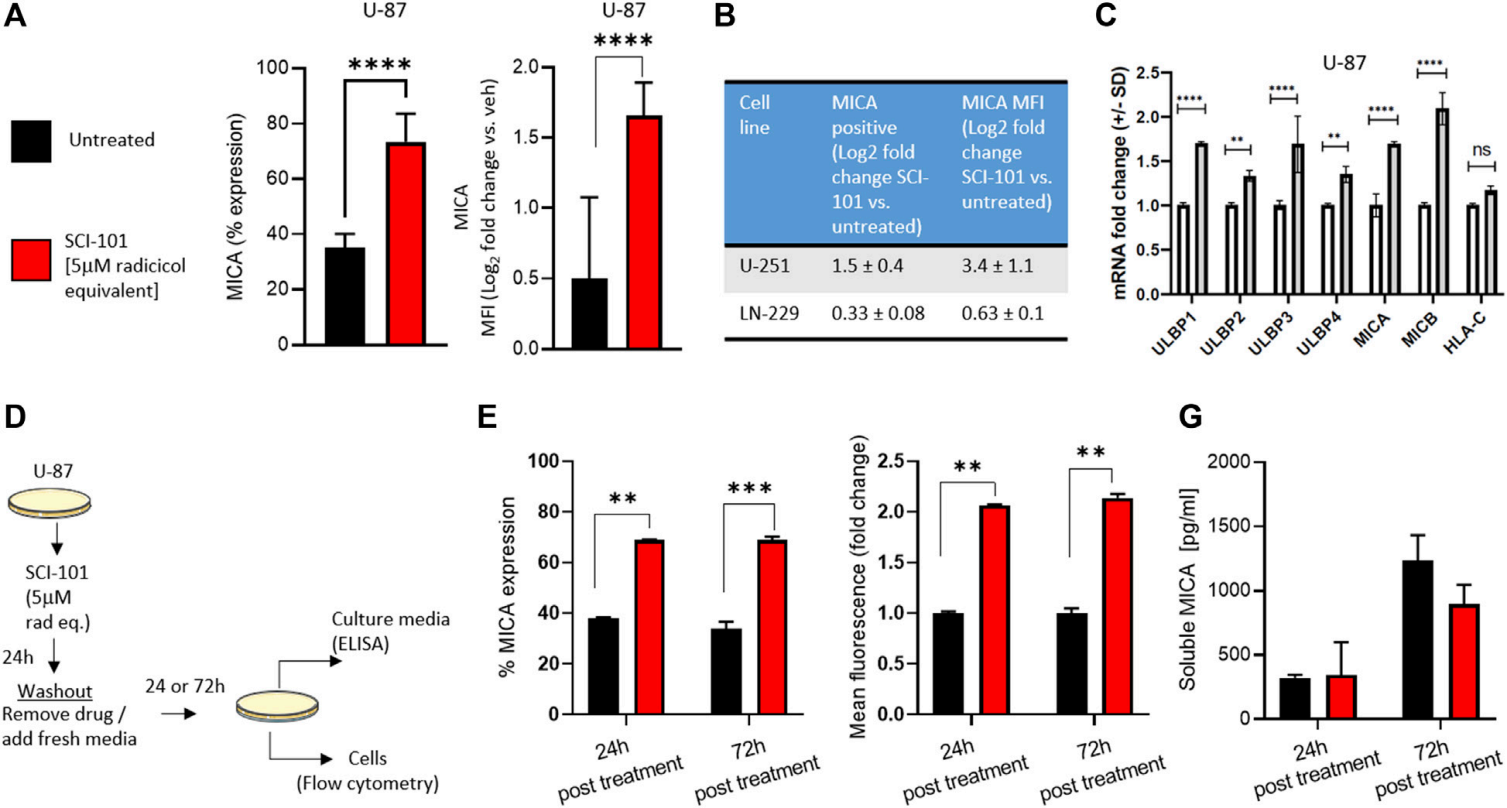
Functional characterization and immunogenic effects of SCI-101 on GBM cells. **(A)** Flow cytometric analysis of MICA expression and mean fluorescent intensity (MFI) in U-87 GBM cells exposed to SCI-101 for 72 h (5 μM). ****p<0.0001 by Student’s t-test. N>4 in biological replicate. **(B)** Table quantifying MICA protein expression and MFI in U-251 and LN-229 GBM cells exposed to SCI-101 for 72 h. Values shown are the log_2_ fold change between treated and untreated conditions. N>3 in biological replicate of each cell line. **(C)** Graphs quantifying mRNA expression for the indicated genes as determined by quantitative PCR. Data are normalized to the GAPDH housekeeping gene. ****p<0.0001, **p<0.01, ns = not significant. **(D)** Schematic illustration of the drug washout experimental design. U-87 cells were untreated or exposed to 5 μM of SCI-101 for 24 h followed by drug washout and replaced with clean media. 24 and 72 h after treatment, the cells were analyzed by flow cytometry or ELISA as described in panels **(E, F)**. **(E)** Flow cytometric analysis of MICA expression and MFI in U-87 GBM cells exposed to SCI-101 for 72 h (5 μM) following the schematic in panel **(D)**. **p < 0.01, ***p < 0.001 by Student’s t-test. N=4 in biological replicate. **(F)** Soluble MICA was determined in U-87 cell culture media following the schematic in panel **(D)**.

We previously reported that radicicol and radicicol-formulated nanoparticles optimally induce the expression of the NKG2D ligands MICA/B and MULT-1 in human and murine cancer models, respectively, compared to other pharmaceutical inhibitors of Hsp90 (Smalley et al., 2020). This has the effect of reversing immunosuppression and improving activity of endogenous and aNK cells (Smalley et al., 2020). We wanted to test the same hypothesis using drug naïve GBM models. First, we determined that SCI-101 induced the expression of MICA/B on U-87 cells (increase from 35 to 74% average expression in untreated and SCI-101–treated cells, respectively) while also increasing the expression density nearly twofold based on flow cytometry data (Figure 3A). Indeed, expression positivity and fluorescence intensity increased in U-251 and, to a lesser degree, LN-229 cells (Figure 3B). We then explored the expression of the ULBP ligand family and HLA-C, which are NKG2D ligands known to augment NK cell activity and tumor immunity *in vivo* (Sutherland et al., 2006). Indeed, the expression of the ULBP family of ligands was augmented with SCI-101 relative to untreated controls, while HLA-C was insignificantly different (Figure 3C). Next, we wanted to evaluate whether SCI-101 could sustain immunogenic activity after drug washout for up to 3 days. We performed two experiments using a drug washout protocol and endpoint evaluation 24 and 72 h later (Figure 3D). Firstly, we determined that SCI-101 sustains the induction of MICA/B-positive cells and MFI, which remains consistent over time (Figure 3E). Next, we asked whether shedding of MICA/B was increased in the drug treatment groups at either time point, which can affect tumor recognition by NK cells (Ferrari de Andrade et al., 2018). Using ELISA, we confirmed that shedding of MICA/B is not detectably changed in the supernatant of GBM cell culture at either 24 or 72 h following drug washout compared to untreated controls (Figure 3F).

### SCI-101 Augments Anticancer Activity and Tumor Infiltration of NK92-MI Cells in 3D *In Vitro* GBM Models

**FIGURE 4:**
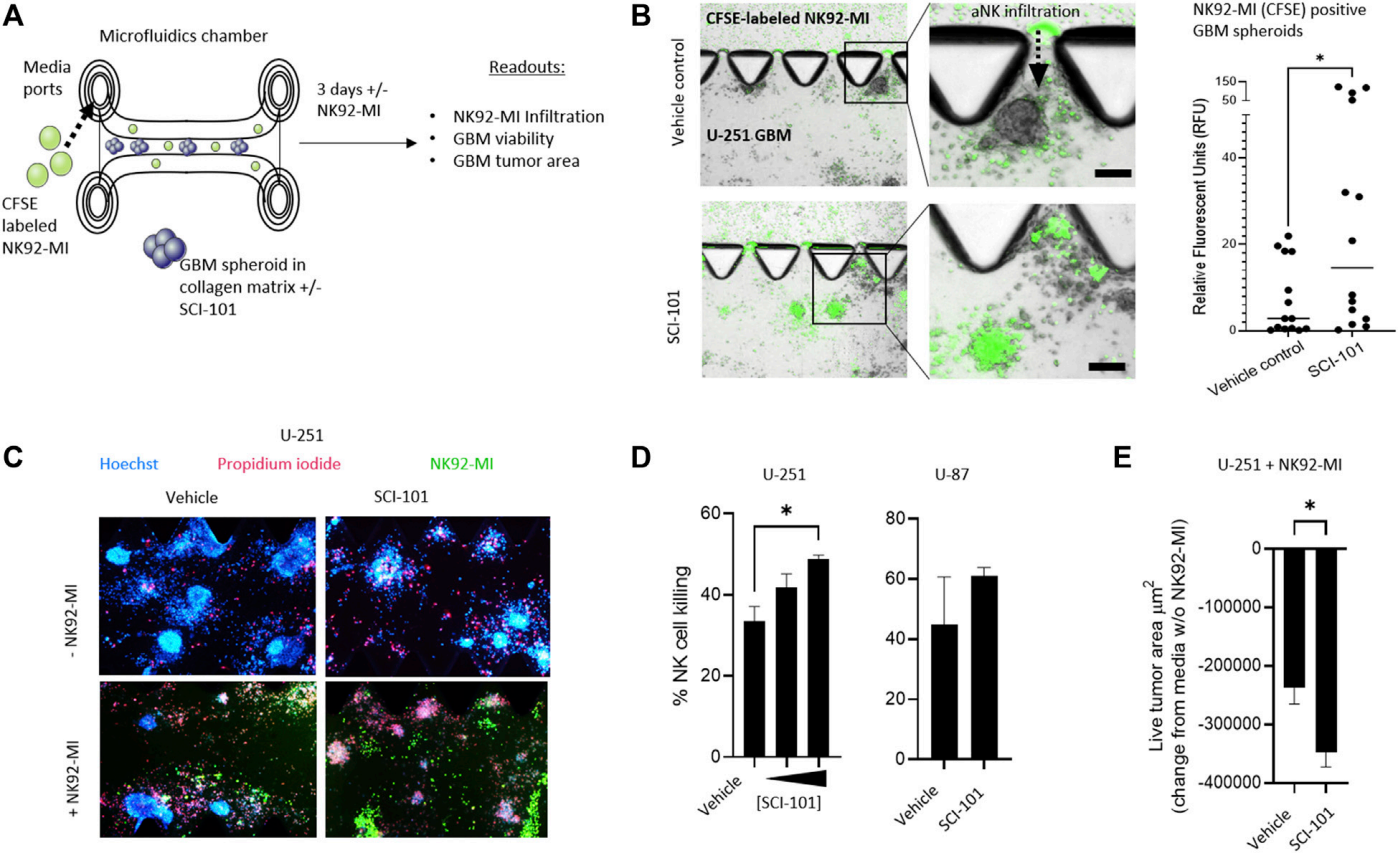
SCI-101 increases NK92-MI tumor infiltration and cell killing in a 3D tumor spheroid model of GBM. **(A)** Microfluidic device and experimental design. GBM tumor spheroids are embedded in a collagen matrix and treated with SCI-101 as described in *Materials and Methods*. CFSE-labeled NK92-MI cells are then introduced to the media ports. Functional and quantitative readouts include infiltration capability of NK92-MI cells and tumor viability and growth. **(B)** Representative fluorescent microscopy overlaying brightfield image of NK92-MI cells (green) in U-251 tumor spheroids. NK cells infiltrate the tumor collagen chamber as indicated by a dashed arrow. Scale bar = 100 μM. The right panel quantifies the relative fluorescence of the CFSE signal in tumor spheroids, as described in *Materials and Methods*. *p<0.05 by the unpaired t-test. **(C)** Representative fluorescent microscope image of U-251 spheroids +/- SCI-101 (10 μM) and NK92-MI (E:T 5:1). **(D)** (left panel) Histogram quantifying normalized NK cell killing in vehicle controls and SCI-101–treated spheroids with 5 μM and 10 μM. (Right panel) NK cell killing in vehicles and SCI-101–treated (10 μM) U-87 spheroids. Values are determined by H/PI image analysis as described in *Materials and Methods*. *p<0.05 by the unpaired t-test. **(E)** Histogram quantifying the live cell area of U-251 spheroids +/- SCI-101 (5 μM) and NK92-MI (E:T 5:1). *p<0.05 by the unpaired t-test.

In a pre-clinical setting, three-dimensional matrix-suspended tumor spheroids provide a closer correlation to tumor physiology, *in situ* and *in vivo*, than 2D cultures (Jensen and Teng, 2020). To test the hypothesis that SCI-101 can augment the activity of aNK cells in GBM, we first deployed a 3D microfluidic tissue culture platform which leverages collagen matrices and tumor spheroids that facilitate *ex vivo* testing of anticancer drugs (Jenkins et al., 2018). Here, we introduced a non-clinical NK cell line, NK92-MI, which is activated in response to NKG2D receptor engagement (Suck et al., 2016) as a tool to investigate the effect of SCI-101 on NK cell infiltration into the tumor spheroid compartment as well as induction of tumor death and release of immune cytokines (Figure 4A). First, we determined that NK92-MI cells are significantly more capable of infiltrating the tumor-containing collagen matrix compartment of SCI-101–treated U-251 GBM spheroids compared to the untreated vehicle control group, which was determined by quantifying the fluorescence intensity of CFSE-labeled NK92-MI cells in tumor spheroids (Figure 4B). Next, we used Hoechst–PI staining to detect the level of NK92-MI cell–induced tumor killing and observed a direct correlation between the dose of SCI-101 and the killing effect of NK92-MI cells in multiple GBM cell lines (Figure 4C) as well as diminishing the residual live tumor spheroid area (Figures 4D,E). These data are consistent with 2D tissue culture experiments (Supplementary Figure 3).

### Generation and Characterization of GTA002, a Clinical Grade aNK Cell

**FIGURE 5:**
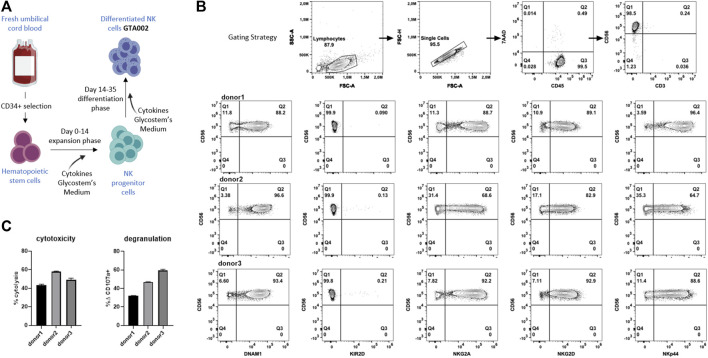
Generation and characterization of GTA002 NK cells using the Glycostem uNiK^™^ process. **(A)** Schematic overview of the expansion and differentiation processes of GTA002 NK cells. **(B)** Representative gating strategy for flow cytometry–based characterization of GTA002 NK cells. GTA002 NK cells were gated on CD45^+^/7-AAD^−^ cells. NK cell receptor expressions are shown below from three different GTA002 NK cell donors at day 35. **(C)** Functionality of GTA002 NK cells was determined by cytotoxicity and degranulation assays against K-562 in a 5 h co-culture assay, with E:T of 1:1. Data are shown as the mean ± SEM of technical triplicates.

Next, we wanted to investigate the clinical translatability and synergistic effect of SCI-101 with aNK cells and test the effect of NKG2D ligand expression on GBM cell lines. To test this, we developed a translational model with clinically deployed aNK cells. Using the uNiK^™^ platform developed by Glycostem Therapeutics B.V., umbilical cord blood–derived CD34^+^ hematopoietic stem cells were expanded and differentiated into aNK cells, termed “GTA002,” in a completely closed system at GMP grade, being a truly off-the-shelf product, due to the final formulation as a cryopreserved product (Spanholtz et al., 2011; Spanholtz et al., 2010). For this specific study, three batches of GTA002 NK cells were cultured in a small scale, mimicking Glycostem’s clinical grade uNiK^™^ culture process (Figure 5A). The culture resulted in highly pure CD56^+^/CD3^-^ GTA002 NK cells (Figure 5B). GTA002 NK cells are characterized by high expression of activating receptors NKG2D, DNAM-1, and NKp44 as well as inhibitory receptor NKG2A and low expression of KIR2D (Figure 5B), which is similar to the phenotype previously published (Spanholtz et al., 2010). A standard 5 h flow-based cytotoxicity assay against K-562 was performed at a low effector:target ratio of 1:1 to assess the functionality of GTA002 NK cells. Killing percentage ranged from 43.3 to 58.0% and degranulation varied from 31.8 to 59.5% for three different GTA002 batches, exhibiting high levels of anti-tumor responses (Figure 5C).

### SCI-101 Boosts GTA002 NK Cell Killing in GBM Models, *In Vitro*

**FIGURE 6:**
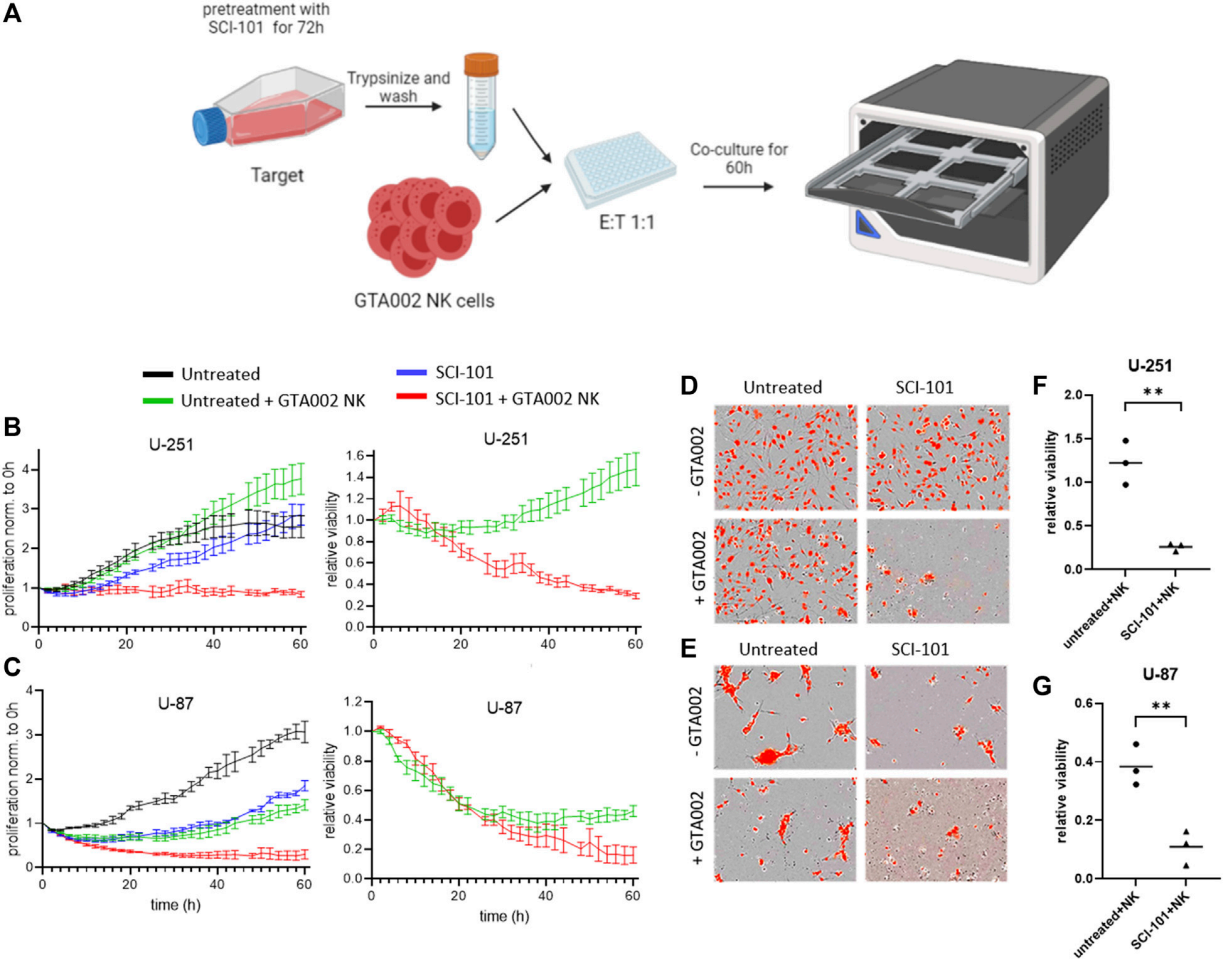
SCI-101 treatment augments glioblastoma susceptibility to GTA002-mediated killing. **(A)** U-87 or U-251 cells pre-treated for 72 h with 5 μM SCI-101 or left untreated were co-cultured with GTA002 cells at an E:T ratio of 1:1 for 60 h in Incucyte. **(B, C)** Cell proliferation traces normalized to time point 0 h and to the relative viability of U-251 **(B)** and U-287 **(C)** are shown as the mean ± SEM of technical quadruplicates for one GTA002 donor. **(D, E)** Representative images of U-251 **(D)** and U-287 **(E)** at t=60 h. Tumor cells are shown with red nuclei. **(F, G)** Statistical analysis of relative viability was performed using the nested t-test at t=60 h for U-251 (F, p=0.0029) and U-287 (G, p=0.0078). Data are shown as the mean ± SEM of n=3 GTA002 donors with ** p<0.01.

Finally, we tested the hypothesis that SCI-101–treated residual GBM cells are more susceptible to killing by GTA002, potentially due to the upregulation of immunogenic phenotypes. Following the schematic in Figure 6A, which mimics a clinical scenario for sequential drug–aNK cell combination therapy, we treated GBM cells with SCI-101 followed by drug washout prior to exposure to GTA002 NK cells (Figure 6A). Consistent with the findings above, a sustained inhibitory effect on cell proliferation beyond 60 h was observed for U-87 after drug withdrawal, while U-251 recovered their baseline proliferation after approximately 50 h (Figure 6B). Importantly, pre-treatment with SCI-101 resulted in differences in NK cell killing susceptibility of both U-251 and U-87. While GTA002 NK cells showed a limited capacity to kill untreated U-251 cells, SCI-101–treated U-251 cells were significantly more sensitive to GTA002 killing (Figure 6B). These findings were consistent with those of U-87 showing a clear separation in curves for SCI-101 + GTA002 NK compared to either GTA002 or SCI-101 alone (Figure 6C). These findings were visually evident under fluorescent microscopy after treatment (Figures 6D,E), and quantification from all three donors showed a statistically significant difference in relative viability after 60 h of co-culture with GTA002 compared to the untreated control GBM cells (*p* < 0.01, Figures 6F,G). Together, this evidence suggests that SCI-101 induces both anticancer and immunoregulatory effects that, when combined with NK cell therapy, synergize the killing potential of clinically deployed aNK cells including GTA002. This effect is likely due to the upregulation of activating ligands on the residual tumor cells. These results provide the basis to further explore the sequential anticancer effect of SCI-101 followed by GTA002 NK cell therapy for GBM in *vivo* models.

## Discussion

We describe a new therapeutic paradigm for cancer and GBM, which combines the use of rationally bioengineered anticancer drugs that boost immunogenic properties of cancer cells in schedule with adoptive cell therapy to ablate residual resistant populations. While approaches to enhance NK cell anti-tumor responses have been developed, including indirect activation of NK cell–mediated antibody-dependent cellular cytotoxicity (ADCC) via engagement of FcγRIIIa/CD16 by CD20 monoclonal antibodies (mAbs) (Tobinai et al., 2017), bispecific NK cell engagers (Bartlett et al., 2020), mAbs that block inhibitory NK receptors (Vey et al., 2018), and mAbs that prevent shedding of NK cell–activating ligands expressed on target cells (Ferrari de Andrade et al., 2018), here we present a novel paradigm that leverages bioengineering principles to improve BBB penetration and the biological activity of the Hsp90 pathway to subsequently improve the activity of clinically deployed adoptive and aNK cell therapies. Indeed, while others have shown combined activity of Hsp90 inhibitors on endogenous NK cells (Fionda et al., 2009), such a “synthetic lethal” pair that combines drug/cell therapy has not been previously described, to the best of our knowledge.

Hsp90 inhibitors have been clinically tested, which generally failed for many solid cancers. These failures tend to be associated with resistance, feedback activation, and redundant signaling pathways among other limitations (Whitesell et al., 2012) including toxicity and bioavailability in the clinic (Neckers and Workman, 2012). Furthermore, Hsp90 inhibitors show anticancer potential (Sauvageot et al., 2009), and brain penetrating analogs continue to be developed for high-grade gliomas (Chen et al., 2020). However, these drugs will continue to be limited by resistance, which is not likely driven through multi-drug–resistant proteins (Sharp et al., 2007). In contrast, we believe our approach is different for two main reasons: Firstly, SCI-101 is constructed in a closed, conjugated lipidic formulation that uses PEGylated residues to confer longer systemic circulation and improved bioavailability, which limits the toxic side effects associated with Hsp90 inhibition in off-target tissue (Craig et al., 2020). Secondly, we exploit Hsp90 resistance mechanisms, rather than become limited by them, which manifest in surviving cells as stress-induced expression of ULBP and MICA/B biomarkers. Indeed, we observed that these activated biomarkers can confer NKG2D-driven aNK cell activity. More advanced *in vivo* studies are warranted to extrapolate the potential side effects, efficacy, and pharmacodynamic/pharmacokinetic relationship of SCI-101 with aNK cells.

Numerous engineering approaches are being developed to augment BBB penetration of therapeutics. However, there is little consensus on the physiochemical constraints that govern “optimal” BBB penetration and drug delivery for GBM. For example, some studies report better active passage through the BBB with neutral structures (Wiwatchaitawee et al., 2021), while anionic nanoparticles show dose-dependent effects on BBB penetration (Lockman et al., 2004). On the contrary, cationic drug vehicles can facilitate brain uptake by adsorptive-mediated transcytosis or endocytosis (Vieira and Gamarra, 2016) (Joshi et al., 2014), while amphiphilic nanoparticles can take advantage of active and passive accumulation (He et al., 2017). Other studies suggest cationic compounds are better able to bind to the negatively charged plasma membrane of the endothelial cells by electrostatic interactions (Vorbrodt, 1989). The lack of consensus for “optimal” formulations implies that efficacy will be drug- and formulation-dependent, and iterations on physiochemical properties should be evaluated on a drug vehicle-by-drug vehicle basis.

NK cell–based therapies are one of the promising candidates in the development of advanced cancer immunotherapies with a diverse source including induced pluripotent stem cells (iPSCs) (Knorr et al., 2013), peripheral blood NK cells from healthy donors, NK cells from umbilical cord blood (Dolstra et al., 2017), and stem cells from cord blood as well as from human cell lines such as NK92, reviewed by Liu et al. (2021). Moreover, additional genetic modifications for introduction of antigen-specific receptors, such as chimeric antigen receptors (CARs) (Uherek et al., 2002) or T-cell receptors (TCRs) (Mensali et al., 2019; Parlar et al., 2019), or overexpression of activating receptors (Sayitoglu et al., 2020), are being explored in an attempt to further enhance the anti-tumor efficacy of NK cell therapies. We hypothesize that the combinatorial use of SCI-101 is not limited to one class of NK cell therapies, but rather it would provide a synergistic advantage to each “class” of NK cell therapies by contributing to the enhancement of fundamental activating signaling input through interaction of NKG2D and its ligands which are upregulated upon SCI-101 treatment (Figure 3). Importantly, tumors including GBM (Chitadze et al., 2015) often secrete NKG2D ligands, for example, through cleavage by ADAM10 and ADAM17 molecules, which often results in decreased surface expression of NKG2D on NK cells and reduced NKG2D-mediated anti-tumor response (Waldhauer and Steinle, 2006; Waldhauer et al., 2008; Liu et al., 2010). Additionally, increased levels of soluble MICA are associated with poor prognosis and disease progression for various cancer types (Holdenrieder et al., 2006; Rebmann et al., 2007). Nevertheless, in this study, we did not observe an increase in the secretion of NKG2D ligand MICA/B, even though SCI-101 treatment significantly increased the expression of NKG2D ligands, depicting the potential safety of SCI-101 in terms of antigen shedding and tumor escape as well as efficiency in triggering enhanced NK cell–mediated anti-tumor responses. Overall, we have tested the synergistic effects of SCI-101 with NK92 and cord blood–derived GTA002 NK cells. In future studies, it will be important to test how CAR-NK and iPSC-derived NK cells are influenced by pre-treatment of tumors with SCI-101. The optimal indications and biomarkers to predict synergistic benefit of SCI-101 treatment in combination with all the different NK cell therapy platforms still need to be identified.

Together, these data provide a novel therapeutic paradigm for combination drug/cell therapy for GBM. The data presented here, *in vitro*, provide merit to the hypothesis for *in vivo* activity and testing toward clinical development.

## Data Availability

The original contributions presented in the study are included in the article/Supplementary Materials, further inquiries can be directed to the corresponding authors.
