# Supplementary material for: Boosting Natural Killer Cell Therapies in Glioblastoma Multiforme Using Supramolecular Cationic Inhibitors of Heat Shock Protein 90
Source: Front Mol Biosci. 2021 Dec 1;8:754443. doi: 10.3389/fmolb.2021.754443 (PMC8673718; doi:10.3389/fmolb.2021.754443)
Supplement: Supplementary file 1 [file DataSheet1.PDF]

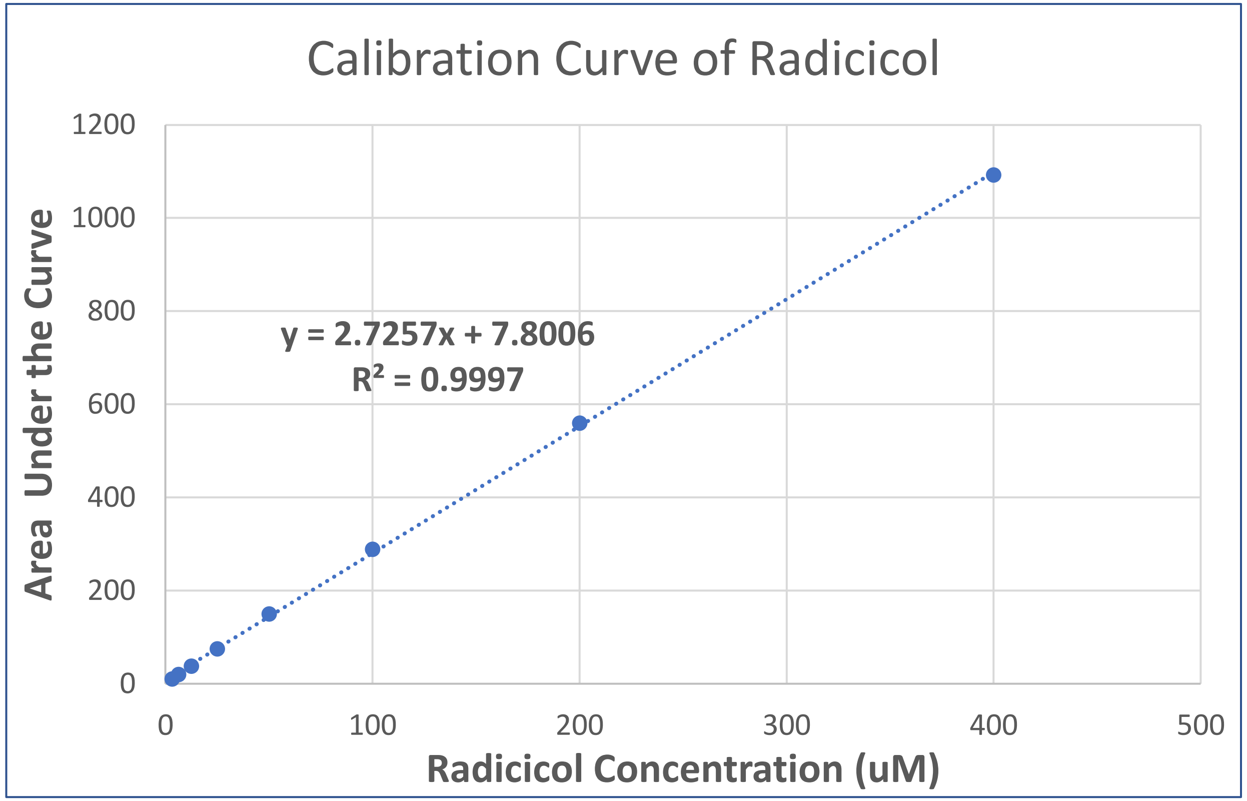

Suppl. Fig 1: LC/MS-MS calibration curve determined for Radicicol

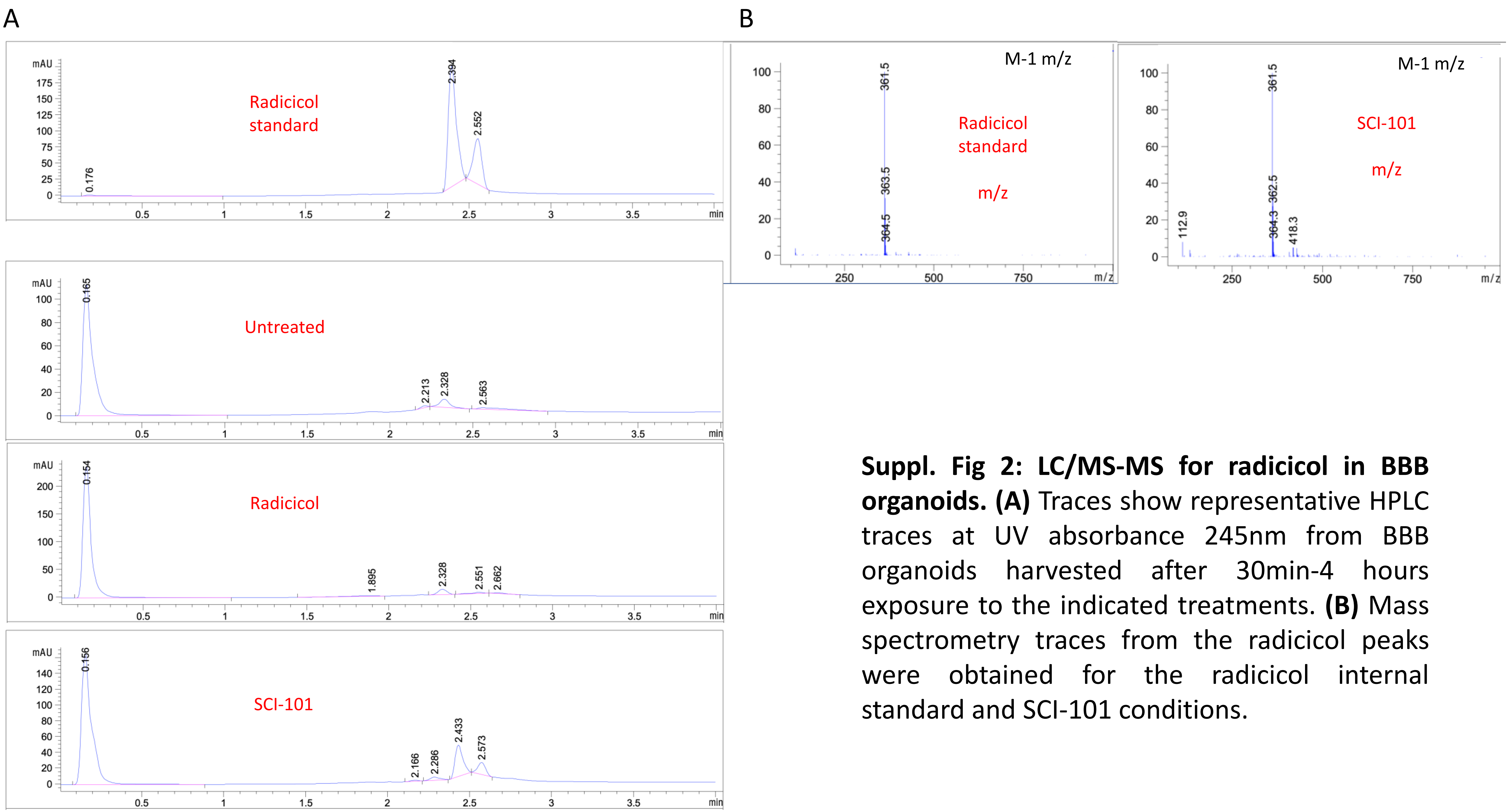

Suppl. Fig 2: LC/MS-MS for radicicol in BBB organoids. (A) Traces show representative HPLC traces at UV absorbance 245nm from BBB organoids harvested after 30min-4 hours exposure to the indicated treatments. **(B)** Mass spectrometry traces from the radicicol peaks were obtained for the radicicol internal standard and SCI-101 conditions.

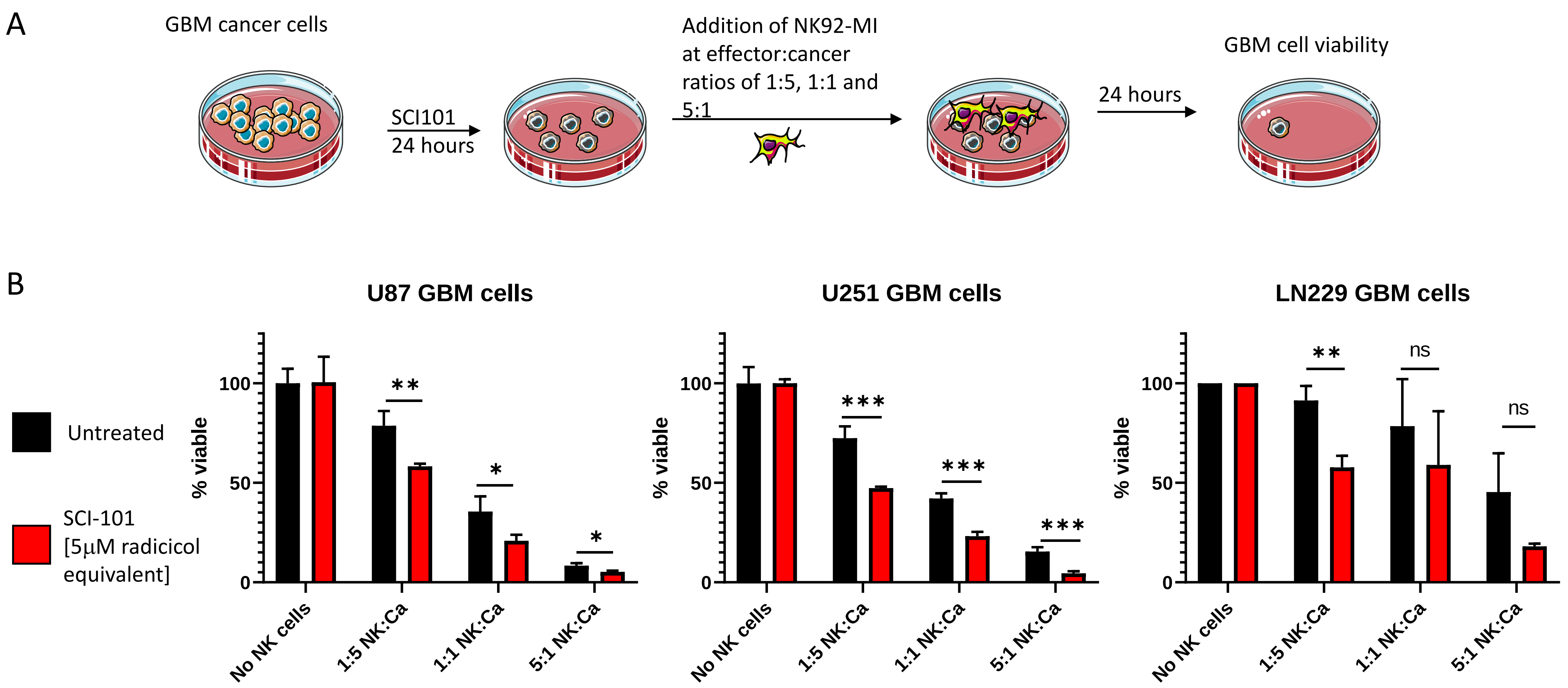

Suppl. Fig 3: SCI-101 activates NK92-MI aNK, in vitro

(A) Schematic of experimental protocol. GBM cells were exposed to vehicle or SCI-101 for 24 hours, washed and exposed to NK92 (in presence of IL-2) at the indicated concentration ratios of NK to cancer (NK:Ca). **(B)** Graphs quantify the % viable determined by CCK-8 assay (viability). Data for each treatment condition were normalized to a 'no NK92' condition for either untreated control or SCI-101 treated. ***p<0.001, **p<0.01, *p<0.05, n.s. not significant determined by student's t-test.
